# Association of Masticatory Function With Mortality in Older Adults

**DOI:** 10.1016/j.identj.2025.103901

**Published:** 2025-09-15

**Authors:** Xindi Wei, Longfei Zhuang, Xiao Zhang, Ke Deng, Ruiying Chen, Beilei Liu, Hongchang Lai

**Affiliations:** a Department of Oral and Maxillofacial Implantology, Shanghai Ninth People's Hospital, Shanghai Jiao Tong University School of Medicine, College of Stomatology, Shanghai Jiao Tong University, National Center for Stomatology, National Clinical Research Center for Oral Diseases, Shanghai Key Laboratory of Stomatology, Shanghai Research Institute of Stomatology, Shanghai, China; b Huishang Dental Clinic, Shanghai, China; c Division of Periodontology and Implant Dentistry, The Faulty of Dentistry, The University of Hong Kong

**Keywords:** Functional tooth unit, Mortality, Masticatory function, Public health

## Abstract

**Introduction and aims:**

The world is rapidly ageing. Tooth loss, the consequence of various age-related oral diseases, leads to decreased chewing function and has emerged as a significant public health concern. The aim of this study was to investigate the association between masticatory function and mortality in older adults.

**Methods:**

Data from the National Health and Nutrition Examination Survey (NHANES) 2009-2018 were analysed. Mortality details were obtained from the National Death Index (NDI). Chewing capacity was determined by the number of functional tooth units (FTUs) that was defined as pairs of opposing natural and artificial teeth in the premolar and molar regions. Weighted Cox proportional hazards models were employed to assess the relationship between FTU and mortality risk. Propensity score matching (PSM) analyses and subgroup analyses were conducted to further assess the association between FTU and mortality.

**Results:**

5,780 individuals aged 60 and above were involved in this study. The risk of all-cause mortality (HR = 2.4, 95%CI 1.8-3.3) was higher for participants with 0 ≤ FTUs ≤ 3 compared to those in the 10 ≤ FTUs ≤ 12 group. After PSM, a significant increase in the risk of all-cause mortality (HR = 1.8, 95%CI 1.4-2.2) was observed in the 0 ≤ FTUs ≤ 3 group compared to the reference group. Subgroup analyses revealed consistent results across all subgroups.

**Conclusions:**

The findings revealed an association between impaired masticatory function and an increased risk of all-cause mortality among older adults.

**Clinical Relevance:**

Study findings show that maintaining and enhancing oral function may help to promote healthy longevity. They also offer guidance for decision-making among older adults and their caregivers.

## Introduction

Ageing is a natural biological process that significantly increases the risk of various health complications.^1^^,^^2^ Advanced body age is correlated with leukocyte telomere lengths and mortality risk, and can predict survival time.^3^^,^^4^ The global population is experiencing rapid ageing, as indicated by epidemiological studies estimating that 1 out of 10 persons in the world is over 60 years old, with predictions suggesting that the ageing population could rise to 22% by 2050.^5^ In the United States, 10%-15% of the population was over 65 years old in 2010.^6^ Merely controlling traditional risk factors, such as lifestyle choices and mental well-being,^7^^,^^8^ is insufficient for reducing the risk of mortality and extending human lifespan. Therefore, it is essential to identify additional modifiable factors to effectively address the pressing public health concern of improving population longevity.

Oral health is an important component of overall well-being, quality of life and potentially healthy ageing. With a significant prevalence worldwide, oral diseases affect more than 3.5 billion people.^9^ For instance, nearly 60% of the US adults aged 65 years or older suffer from periodontal disease.^10^ However, the correlation between oral diseases and mortality in older adults is often overlooked. Untreated oral diseases can have severe repercussions, adversely impacting masticatory function, which is a crucial aspect of healthy ageing.^11^^,^^12^ A study conducted on Chinese adults revealed that a higher count of natural teeth among older adults is correlated with a decreased risk of mortality.^13^ However, the teeth number cannot accurately reflect masticatory function impairment. While some studies have employed self-reported chewing ability as a proxy for masticatory function, this approach is relatively subjective.^14^ In fact, chewing ability is substantially reduced only when there's absence of opposing premolar and molar pairs. Functional tooth units (FTUs), defined as pairs of opposing teeth in the premolar and molar areas, serve as a more reliable indicator and can be easily calculated.^15^ Several studies have linked impaired masticatory function with increased risk of mortality.^16^^,^^17^ Notably, a recent prospective study further identified an association between 0 ≤ FTU ≤ 4 and higher risk of all-cause mortality, highlighting that phenotypic age acceleration and frailty index mediated this relationship.^18^ However, previous studies have not comprehensively investigated the effects of FTU on all-cause mortality based on older adults across the United States.

The objective of this study was to investigate the association between FTU and all-cause mortality using data from the National Health and Nutrition Examination Survey (NHANES) spanning 2009-2018.

## Methods

### Study design and population

This study used data from the NHANES, a nationally representative health survey conducted by the Centers for Disease Control and Prevention's (CDC) National Center for Health Statistics (NCHS) in the United States (https://www.cdc.gov/nchs/nhanes/index.htm). Mortality details were obtained from the National Death Index (NDI) (https://www.cdc.gov/nchs/data-linkage/mortality-public.htm). The NHANES and NDI are linked by matching SEQN. These databases are openly available for download from their respective websites. The survey targets the civilian non-institutionalised resident population. Questionnaires were administered in the participants' homes followed by a standardised examination performed in a specially equipped mobile examination center (MEC), and all the participants provided written informed consent.^19^ In this cohort study, 5 cycles of survey data obtained from NHANES 2009-2018 were used. This study follows the strengthening the reporting of observational studies in epidemiology (STROBE) guidelines (Supplementary Table 1).^20^

**Fig. 1:**
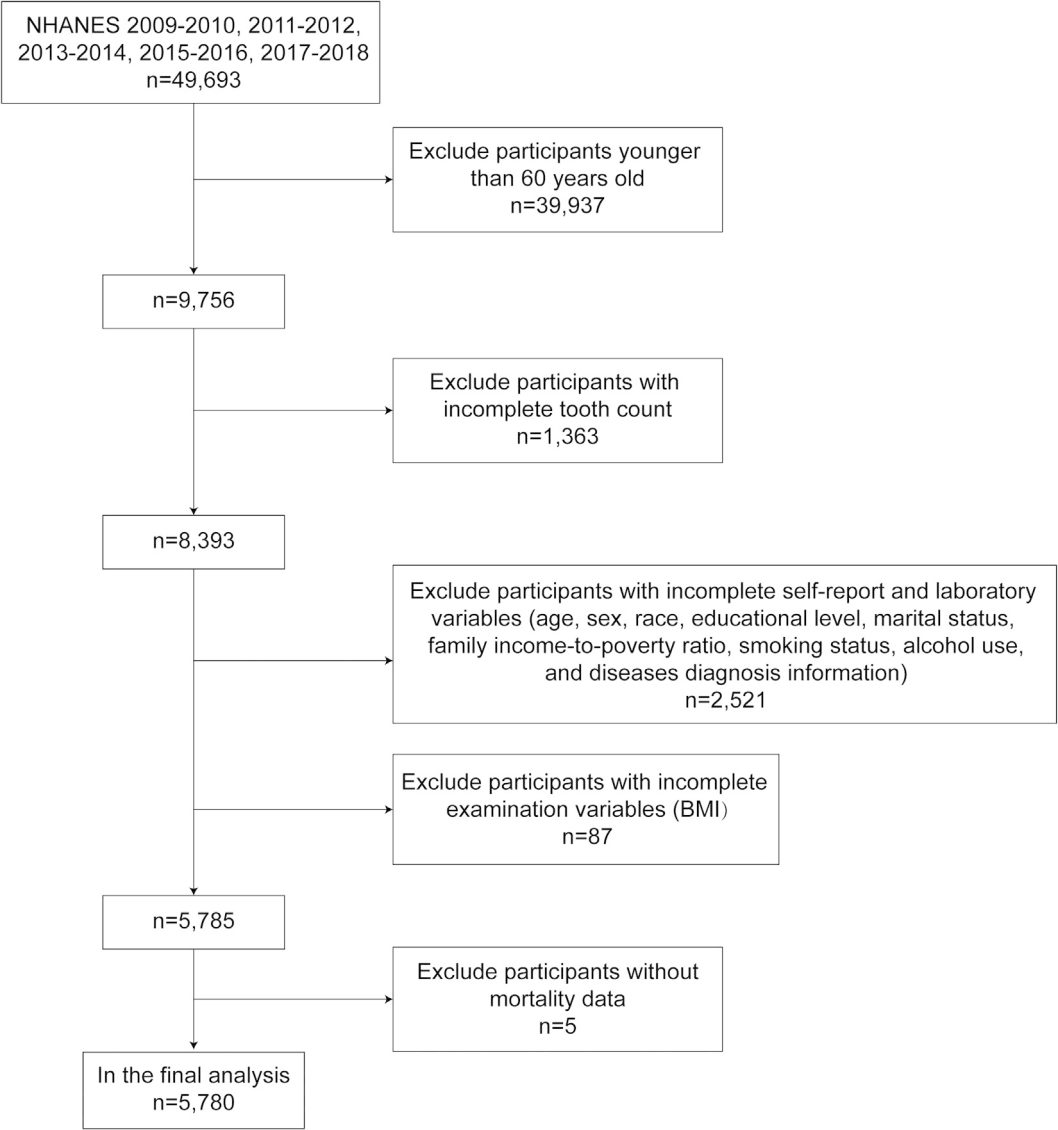
Flowchart of the process for the selection of eligible participants.

The sample selection process is depicted in Figure 1, and an analytical sample of 5,780 was obtained from the combination of the 5 NHANES cycles. Only participants with complete data on dental examination, follow-up and relevant covariates were included in the analyses. Participants who failed to meet these criteria were excluded from the analyses.

### Independent variable

FTU was the independent variable in this study. The number of FTUs was defined as pairs of opposing natural and artificial teeth in the premolar and molar regions, and the third molars were excluded. Two opposing premolars are described as 1 FTU, and 2 opposing molars are defined as 2 FTUs. Because of the data limitation in NHANES, the FTUs were determined based on the presence of teeth in this study.^21^ The Oral Health – Dentition section was used to assess the presence of teeth and calculate the FTU.

### Dependent variable

The main outcome was mortality, and the mortality status was identified through the linkage between NHANES and the death certificate records from the NDI which was conducted by the NCHS in the United States (https://www.cdc.gov/nchs/data-linkage/mortality.htm). The MORTSTAT and ucod_leading variables were used as the status of death, and the PERMTH_INT variable was used as the follow-up time. All-cause and cause-specific mortality was included in this study to evaluate the impact of impaired chewing ability on the survival status of individuals.

### Covariates

The covariates were selected based on a literature review and clinical experiences. All the participants provided information on age (60-65 years, 66-70 years, 71-75 years, 76-80 years), sex (male, female), race (Mexican American, Non-Hispanic white, Non-Hispanic Black, Other Hispanic/Other race), educational level (less than 9th grade, 9-11th grade, high school graduate/GED or equivalent, some college or AA degree, college graduate or above), marital status (married, living with partner, widowed, divorced, separated, never married), family income-to-poverty ratio (<1, ≥1), smoking (non-smokers, former smokers, current smokers), drinking (yes, no), kidney disease (yes, no), coronary heart disease (CHD) (yes, no), chronic bronchitis (yes, no) and cancer (yes, no) on the survey questionnaires. Individuals aged 80 and above were top-coded as 80 years of age in NHANES. Smoking status was assessed as non-smokers (smoked <100 cigarettes in their life), former smokers and current smokers. Drinking was defined as having alcohol at least 3 times per week on average in the past 12 months. Body mass index (BMI) was calculated using weight (kg)/height (m^2^). Obese was defined as a body mass index (BMI) ≥ 30 kg/m^2^, and overweight was defined as 25 kg/m^2^ < BMI < 30 kg/m^2^.^22^ Participants were identified as suffering from hypertension (yes, no) if one of the following criteria was met: (a) had a systolic blood pressure ≥ 140 mmHg or a diastolic blood pressure ≥ 90 mmHg; or (b) a self-reported diagnosis of hypertension. Participants were identified as having diabetes (yes, no) if they met any of the following criteria: (a) had a hemoglobin A1C concentration ≥ 6.5%, (b) had a fasting plasma glucose level ≥ 126 mg/dL, (c) self-reported use of antidiabetic medications or (d) self-reported diagnosis of diabetes. Model design and confounders were visualised using a directed acyclic graph (DAG), which is shown in Supplementary Figure 1.

### Statistical analysis

Weighting was taken into account in this study. By weighting the sample data, analysts were able to generate estimates of the statistics as if the entire eligible population had been surveyed. WTMEC2YR (examination weights) or WTSAF2YR (fasting subsample weights) divided by 5 was chosen as the sample weight for this study. In descriptive statistics, the Chi-squared test was used to compare the percentages of categorical variables among distinct FTU groups. The weighted univariate and multivariate Cox proportional hazards models were adopted to evaluate the hazard ratios (HR) and 95% confidence intervals (CI) pertaining to the association between FTU and mortality. The proportional hazards assumption was evaluated using the cox.zph() function of the survival package. The mortality rates among the distinct FTU groups were shown in Kaplan-Meier curves. To explore potential non-linear relationships between FTU and all-cause mortality, restricted cubic splines (RCS) were deployed. A 1:1 propensity score matching (PSM) analysis was conducted to balance the differences between two groups, which adjusted for age, sex, race, education level, marital status, family income-to-poverty ratio, smoking status, alcohol use, obesity, diabetes, hypertension, kidney disease, CHD, chronic bronchitis and cancer. The data after PSM were then analysed by weighted Cox regression to confirm the association between FTU and mortality, and no covariate was adjusted in the model. Subgroup analyses, stratified by age, race, sex, obesity and disease histories, were conducted to explore the association between FTU and all-cause mortality in distinct situations. *P* for interaction was calculated via the likelihood ratio test.

To control for confounders, three weighted Cox regression models were constructed to eliminate the influence of covariates. No variables were adjusted in Model 1. Model 2 accounted for age, sex, race, education level, marital status and family income-to-poverty ratio. Model 3 further incorporated adjustments for smoking status, alcohol use, obesity, diabetes, hypertension, kidney disease, CHD, chronic bronchitis and cancer. Covariates in the models were selected based on existing scientific knowledge.^23^

A two-sided *P* < .05 was considered statistically significant in all analyses. All statistical analyses were performed using R 4.3.1 (http://www.R-project.org).

## Results

### Baseline characteristic of study samples

A total of 5,780 NHANES 2009-2018 participants were included in the present study. The baseline characteristics of these participants are shown in Table 1. Significant differences were detected among people with different numbers of FTU in various factors, including age, sex, race, marital status, educational level, family income-to-poverty ratio, obesity, smoking, diabetes, hypertension, kidney disease, CHD, chronic bronchitis and cancer (*P* < .05). For the population included in this study, older individuals exhibited decreased FTUs. The proportion of individuals with 0 ≤ FTUs ≤ 3 was higher among males. Individuals with lower levels of education generally had poorer oral health compared to those with higher education levels. There was a higher prevalence of overweight and obese individuals. Moreover, there were a significant number of patients with diabetes and hypertension, whereas patients with other systemic diseases were relatively fewer. During a median follow-up period of 60 (33-91) months, 960 (16.6%) of the 5,780 participants died.

**Table 1:** Characteristics of the participants (*N* = 5,780).

Characteristics	10 ≤ FTUs ≤ 12	7 ≤ FTUs ≤ 9	4 ≤ FTUs ≤ 6	0 ≤ FTUs ≤ 3
**Age (%)**				
**60-65**	554 (44.8)	321 (41.5)	321 (39.4)	911 (30.8)
**66-70**	283 (22.9)	159 (20.5)	173 (21.2)	645 (21.8)
**71-75**	185 (15.0)	112 (14.5)	151 (18.5)	520 (17.6)
**76-80**	214 (17.3)	182 (23.5)	170 (20.9)	879 (29.7)
**Sex (%)**				
**Male**	640 (51.8)	416 (53.7)	441 (54.1)	1,692 (57.3)
**Female**	596 (48.2)	358 (46.3)	374 (45.9)	1,263 (42.7)
**Race (%)**				
**Mexican American**	138 (11.2)	94 (12.1)	114 (14.0)	268 (9.1)
**Non-Hispanic White**	804 (65.0)	411 (53.1)	352 (43.2)	1,326 (44.9)
**Non-Hispanic Black**	106 (8.6)	126 (16.3)	183 (22.5)	872 (29.5)
**Other**	188 (15.2)	143 (18.5)	166 (20.4)	489 (16.5)
**Education (%)**				
**Less than 9th grade**	60 (4.9)	56 (7.2)	87 (10.7)	425 (14.4)
**9-11th grade**	54 (4.4)	61 (7.9)	95 (11.7)	546 (18.5)
**High school graduate/GED or equivalent**	174 (14.1)	161 (20.8)	191 (23.4)	847 (28.7)
**Some college or AA degree**	364 (29.4)	260 (33.6)	246 (30.2)	801 (27.1)
**College graduate or above**	584 (47.2)	236 (30.5)	196 (24.0)	336 (11.4)
**Marital status (%)**				
**Married**	853 (69.0)	483 (62.4)	461 (56.6)	1,461 (49.4)
**Widowed**	131 (10.6)	110 (14.2)	130 (16.0)	635 (21.5)
**Divorced**	160 (12.9)	108 (14.0)	126 (15.5)	495 (16.8)
**Separated**	18 (1.5)	19 (2.5)	24 (2.9)	97 (3.3)
**Never married**	50 (4.0)	30 (3.9)	45 (5.5)	169 (5.7)
**Living with partner**	24 (1.9)	24 (3.1)	29 (3.6)	98 (3.3)
**Family income-to-poverty ratio (%)**				
**<1**	1,156 (93.5)	689 (89.0)	710 (87.1)	2,315 (78.3)
**≥1**	80 (6.5)	85 (11.0)	105 (12.9)	640 (21.7)
**Obesity (%)**				
**Normal**	324 (26.2)	189 (24.4)	160 (19.6)	724 (24.5)
**Overweight**	482 (39.0)	288 (37.2)	296 (36.3)	1,014 (34.3)
**Obese**	430 (34.8)	297 (38.4)	359 (44.0)	1,217 (41.2)
**Smoke (%)**				
**Current smoker**	57 (4.6)	55 (7.1)	89 (10.9)	634 (21.5)
**Former smoker**	457 (37.0)	325 (42.0)	349 (42.8)	1,366 (46.2)
**Non-smoker**	722 (58.4)	394 (50.9)	377 (46.3)	955 (32.3)
**Alcohol (%)**				
**Yes**	51 (4.1)	41 (5.3)	27 (3.3)	72 (2.4)
**No**	1,185 (95.9)	733 (94.7)	788 (96.7)	2,883 (97.6)
**Diabetes (%)**				
**Yes**	277 (22.4)	222 (28.7)	268 (32.9)	1,075 (36.4)
**No**	959 (77.6)	552 (71.3)	552 (71.3)	1,880 (63.6)
**Hypertension (%)**				
**Yes**	744 (60.2)	545 (70.4)	582 (71.4)	2,252 (76.2)
**No**	492 (39.8)	229 (29.6)	233 (28.6)	703 (23.8)
**Kidney disease (%)**				
**Yes**	40 (3.2)	31 (4.0)	38 (4.7)	38 (4.7)
**No**	1,196 (96.8)	743 (96.0)	777 (95.3)	2,712 (91.8)
**CHD (%)**				
**Yes**	100 (8.1)	65 (8.4)	72 (8.8)	349 (11.8)
**No**	1,136 (91.9)	709 (91.6)	743 (91.2)	2,606 (88.2)
**Chronic bronchitis (%)**				
**Yes**	64 (5.2)	49 (6.3)	52 (6.4)	287 (9.7)
**No**	1,172 (94.8)	725 (93.7)	763 (93.6)	2,668 (90.3)
**Cancer (%)**				
**Yes**	311 (25.2)	190 (24.5)	170 (20.9)	632 (21.4)
**No**	925 (74.8)	584 (75.5)	645 (79.1)	2,323 (78.6)

Abbreviations: FTU, functional tooth unit; CHD, coronary heart disease.

### Association between lower FTU levels and increased mortality risk

The results from the Cox regression analyses on the association between FTU and all-cause mortality are summarized in Table 2. The proportional hazards assumption was met for all variables, with all *P* > .05. In model 1, the risk for all-cause mortality (HR = 4.6, 95%CI 3.5-6.1) increased in the 0 ≤ FTUs ≤ 3 group compared to the 10 ≤ FTUs ≤ 12 group. Survival curve analyses showed a significantly decreased survival rate in the 0 ≤ FTUs ≤ 3 group compared to the 10 ≤ FTUs ≤ 12 group (Supplementary Figure 2A). After adjusting for all confounders, compared to that of the reference group (10 ≤ FTUs ≤ 12), the risk of all-cause mortality (HR = 2.4, 95%CI 1.8-3.3) was still increased for participants with 0 ≤ FTUs ≤ 3. RCS analysis revealed a linear association between FTU and all-cause mortality (for non-linear, *P* > .05) (Supplementary Figure 3). A preliminary exploration was also conducted on the impact of FTU on disease-specific mortality. The results of Cox regression analyses regarding the association between FTU and heart disease mortality are summarised in Supplementary Table 2. After adjusting for all confounders, compared with the reference group (10 ≤ FTUs ≤ 12), the risk of heart disease mortality in participants with 0 ≤ FTUs ≤ 3 was elevated (HR = 2.1, 95%CI 1.2-3.7).

**Table 2:** Association between FTU and all-cause mortality.

	Count (%)	Model 1		Model 2		Model 3	
		HR (95%CI)	*P*	HR (95%CI)	*P*	HR (95%CI)	*P*
10 ≤ FTUs ≤ 12	1,236 (21.4)	Ref		Ref		Ref	
7 ≤ FTUs ≤ 9	774 (13.4)	1.9 (1.3, 2.9)	.001	1.7 (1.1, 2.4)	0.009	1.4 (1.0, 2.1)	.062
4 ≤ FTUs ≤ 6	815 (14.1)	2.5 (1.6, 3.8)	<.001	2.1 (1.4, 3.2)	<0.001	1.8 (1.3, 2.6)	.001
0 ≤ FTUs ≤ 3	2,955 (51.1)	4.6 (3.5, 6.1)	<.001	3.5 (2.7, 4.7)	<0.001	2.4 (1.8, 3.3)	<.001

Note: *P* < .05 indicates statistical significance.

Abbreviations: HR, hazard ratio; CI, confidence interval; Ref: reference; FTU, functional tooth unit; CHD, coronary heart disease.

Note: Model 1—Unadjusted. Model 2—Model 1 additionally adjusted for age, sex, race, education level, marital status and family income-to-poverty ratio. Model 3—Model 2 plus additional adjustment for smoking status, alcohol use, obesity and disease histories (including diabetes, hypertension, kidney disease, CHD, chronic bronchitis and cancer).

PSM analysis was then used to further evaluate the association between FTU and mortality. FTUs were divided into 2 categories for PSM analyses, including 0 ≤ FTUs ≤ 3 and 4 ≤ FTUs ≤ 12. All covariates were not significantly different after PSM (Supplementary Table 2). Survival curve analyses showed a significantly decreased survival rate in the 0 ≤ FTUs ≤ 3 group compared to the 4 ≤ FTUs ≤ 12 group (Supplementary Figure 2B). A significant increase in the risk of all-cause mortality (HR = 1.8, 95%CI 1.4-2.2) in 0 ≤ FTUs ≤ 3 group was observed compared to the reference group (Supplementary Table 3).

### Subgroup analyses by potential effect modifiers

**Fig. 2:**
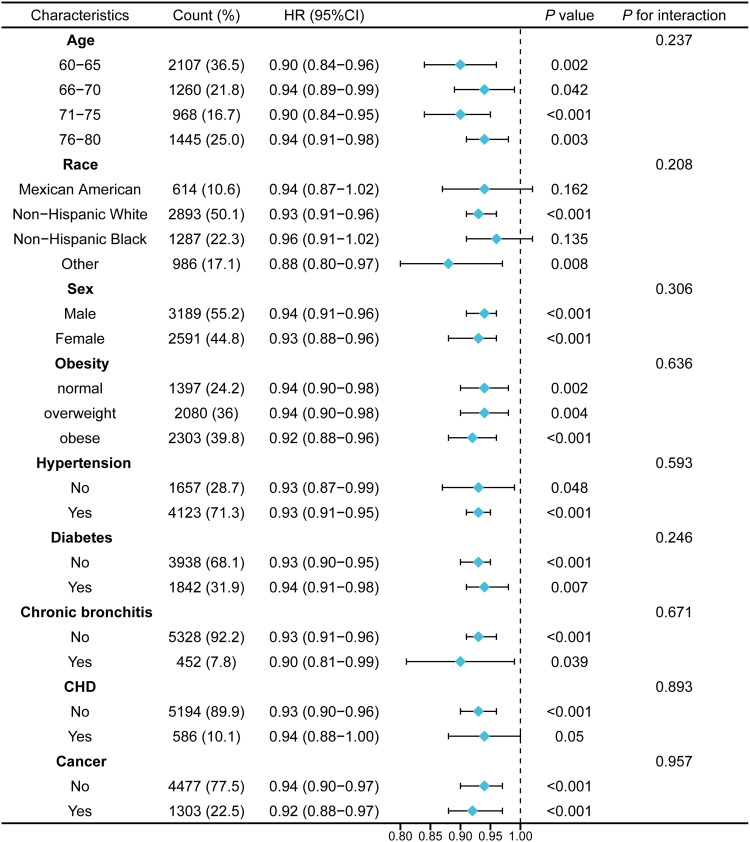
Subgroup analyses by possible effect modifiers for the relationship between FTU and all-cause mortality.

When the FTU was analysed as a continuous predictor, it was negatively correlated with all-cause death (HR = 0.93, 95%CI 0.91-0.95), which corroborated the results of the previous analysis. Subgroup analyses based on age, sex, obesity and disease histories were conducted to assess the reliability of the association between FTU and all-cause mortality. Nearly all subgroups exhibited a negative association between the FTU and the risk of all-cause mortality. No significant interaction was observed in all subgroups (for interaction, *P* > .05), indicating the robustness of the correlation between FTU and all-cause mortality (Figure 2).

## Discussion

This study revealed the correlation between FTU and mortality among older populations through diverse methodologies. The analysis of data from NHANES 2009-2018 revealed a correlation between reduced FTUs and a higher risk of all-cause mortality, indicating that individuals with impaired masticatory function were more susceptible to death. Importantly, the correlation between FTUs and mortality persisted after PSM analysis. Furthermore, this association remained consistent across various subgroups.

Prior evidence has suggested a correlation between masticatory function and mortality. The oral frailty assessed by 6 indicators, including chewing ability, was associated with a 2.2-fold increased risk of mortality.^24^ Studies in Japan and Europe also suggested that poor chewing ability was correlated with mortality in older individuals.^25^^,^^26^ Another study found that the number of FTUs <5 was associated with an increased risk of mortality, with an HR of 1.72 for all-cause mortality, HR of 1.41 for cardiovascular mortality, HR of 1.76 for cancer mortality, and HR of 1.85 for non-cardiovascular and non-cancer mortality.^16^ Additionally, a study conducted in nursing homes in Japan showed that the lack of occluding posterior teeth was correlated with 1-year mortality.^27^ For individuals aged 80 years, masticatory dysfunction was significantly related to mortality.^28^^,^^29^ Even for middle-aged adults who exercised regularly, impaired masticatory function was a risk factor for mortality.^30^ These findings align with the results derived from the Cox regression analyses in this study, and this study further extends the current knowledge by demonstrating that 0 ≤ FTUs ≤ 3 is associated with a higher risk of all-cause mortality in older adults. Even after PSM correction for differences, the association between decreased FTUs and increased risk of all-cause mortality persists. In this study, the HR of mortality reached 2.4 when the number of FTUs was lower than 3, indicating that these older adults may be high-risk individuals warranting more attention.

The mechanistic pathways underlying the relationship between FTU and mortality remain unexplored, but several possibilities have been proposed. Previous studies demonstrated that an insufficient number of FTUs may lead to a preference for easily chewable foods, such as ultra-processed food which is less healthy, thereby resulting in a dietary intake pattern that is related to an increased risk of mortality.^21^^,^^31^, ^32^, ^33^, ^34^, ^35^ Moreover, another study confirmed that tooth loss was related to decreased diet quality and accelerated ageing.^36^ Speculatively, FTU may influence food intake patterns, potentially impacting diet quality and consequently increasing mortality risks. In addition, dental damage can induce trigeminal nerve injury and degeneration of the cholinergic system. The degree of cholinergic dysfunction is positively related to cognitive impairment which may increase the risk of mortality during old age.^37^^,^^38^

The findings of this study contribute to the development of therapeutic approaches to improve healthy longevity. Restoring lost FTUs might serve as a method to reduce the risk of mortality in older adults, which underscores the significance of carrying out oral rehabilitation, such as removable denture restoration and implant restoration.

The strength of this study is that the findings come from a large nationally representative sample of older adults in the United States to investigate the correlation between FTU and mortality, which improved the statistical capacity. Three models adjusted for multiple confounders and PSM analysis were applied to ensure the reliability of the results. However, this study has several limitations. Firstly, more chewing capacity indicators other than FTU, such as masticatory muscle strength, should be used to validate the findings. Secondly, because of the absence of longitudinal FTU data on participants in NHANES, this study was unable to investigate the dynamic relationship between FTU changes over time and changes in mortality risk. Thirdly, despite adjusting for numerous confounding factors, the observed association may still be susceptible to unmeasured or residual confounders.

## Conclusion

In conclusion, this study unveiled an association between impaired chewing capacity and an elevated risk of all-cause mortality among the ageing population. Decreased FTUs, especially 0 ≤ FTUs ≤ 3, heightened mortality risk among this demographic. Nevertheless, it is important to acknowledge that this relationship may be influenced by various confounding factors. These findings highlight the importance of maintaining and improving oral health as a factor that improves healthy longevity. Policy should focus on strategies to improve oral health care access, education and interventions tailored to address the specific needs of older individuals. Further studies based on prospective data should be conducted to verify the findings and understand the potential mechanisms.

## Conflict of interest

None declared.

## Appendix Supplementary materials

Image, application 1

## Footnotes

Supplementary material associated with this article can be found in the online version at doi:10.1016/j.identj.2025.103901.
